# Membrane Bioreactor (MBR) Technology for Wastewater Treatment and Reclamation: Membrane Fouling

**DOI:** 10.3390/membranes6020033

**Published:** 2016-06-15

**Authors:** Oliver Terna Iorhemen, Rania Ahmed Hamza, Joo Hwa Tay

**Affiliations:** Department of Civil Engineering, University of Calgary, Calgary, AB T2N 1N4, Canada; rania.sayedeid@ucalgary.ca (R.A.H.); jhatay@ucalgary.ca (J.H.T.)

**Keywords:** aerobic granulation, extracellular polymeric substances (EPS), membrane bioreactor (MBR), membrane fouling, quorum quenching, soluble microbial products (SMPs), wastewater treatment

## Abstract

The membrane bioreactor (MBR) has emerged as an efficient compact technology for municipal and industrial wastewater treatment. The major drawback impeding wider application of MBRs is membrane fouling, which significantly reduces membrane performance and lifespan, resulting in a significant increase in maintenance and operating costs. Finding sustainable membrane fouling mitigation strategies in MBRs has been one of the main concerns over the last two decades. This paper provides an overview of membrane fouling and studies conducted to identify mitigating strategies for fouling in MBRs. Classes of foulants, including biofoulants, organic foulants and inorganic foulants, as well as factors influencing membrane fouling are outlined. Recent research attempts on fouling control, including addition of coagulants and adsorbents, combination of aerobic granulation with MBRs, introduction of granular materials with air scouring in the MBR tank, and quorum quenching are presented. The addition of coagulants and adsorbents shows a significant membrane fouling reduction, but further research is needed to establish optimum dosages of the various coagulants/adsorbents. Similarly, the integration of aerobic granulation with MBRs, which targets biofoulants and organic foulants, shows outstanding filtration performance and a significant reduction in fouling rate, as well as excellent nutrients removal. However, further research is needed on the enhancement of long-term granule integrity. Quorum quenching also offers a strong potential for fouling control, but pilot-scale testing is required to explore the feasibility of full-scale application.

## 1. Introduction

Membrane bioreactor (MBR) technology has emerged as a wastewater treatment technology of choice over the activated sludge process (ASP), which has been the conventional municipal wastewater technology over the last century. MBR is, in fact, one of the most important innovations in wastewater treatment [1,2,3,4], as it overcomes the drawbacks of the conventional ASP, including large space requirement for secondary clarifiers, liquid–solid separation issues, production of excess sludge, and limitations with removal of recalcitrants [5]. MBRs have been used for both municipal and industrial wastewater treatment and reclamation [6,7,8,9,10]. An MBR is a hybrid of a conventional biological treatment system and physical liquid–solid separation using membrane filtration [11,12,13] in one system. The MBR technology provides the following advantages over ASP: High-quality effluent, higher volumetric loading rates, shorter hydraulic retention times (HRT), longer solid retention times (SRT), less sludge production, and potential for simultaneous nitrification/denitrification in long SRTs [2,5,13,14,15,16]. The inclusion of membranes in the system eliminates the need for secondary clarifiers. The elimination of secondary clarifiers and operation of MBR at a shorter HRT results in significantly reduced plant area requirements. However, the use of MBR technology has disadvantages, including higher energy costs, the need to control membrane fouling problems, and potential high costs of periodic membrane replacement [17].

Membrane fouling remains a major drawback of MBR [2,18,19], as it significantly reduces membrane performances and membrane lifespan, leading to an increase in maintenance and operating costs [12,20]. Membrane fouling in MBRs is attributable to suspended particulates (microorganisms and cell debris), colloids, solutes, and sludge flocs [2]. These materials deposit onto the membrane surface and into the membrane pores, clogging the pores, and leading to a decline in the permeability of the membrane [11]. The heterogeneous nature of suspended solids and active microorganisms in mixed liquor suspended solids (MLSS) makes membrane fouling an inevitable challenge that is difficult to control in long-term MBR applications [12]. Membrane fouling mitigation in MBRs has been one of the key areas of extensive research in order to enhance the wider application of the MBR technology in wastewater engineering.

This paper provides an overview of the fundamentals of membrane fouling and advances in fouling mitigation strategies in MBRs, based on the recent and relevant publications on membrane fouling. The review covers background information on membrane fouling, classes of membrane foulants in MBRs, and a discussion on the factors affecting membrane fouling in MBRs. This is followed by a comprehensive review of the current research trends for membrane fouling control in MBR.

## 2. Membrane Fouling in MBR

According to the International Union of Pure and Applied Chemistry (IUPAC) Working Party on Membrane Nomenclatures, membrane fouling is “the process resulting in loss of performance of a membrane due to the deposition of suspended or dissolved substances on its external surfaces, at its pore openings, or within its pores” [21]. These foulants can be suspended particulates (microorganisms and cell debris), colloids, and solutes in the MLSS [2,14,18]. The physico-chemical interactions that take place between the foulants and the membrane material result in membrane fouling. Failure to appropriately control membrane fouling in MBRs may, in some cases, lead to failure to treat the required design flows [13].

**Figure 1:**
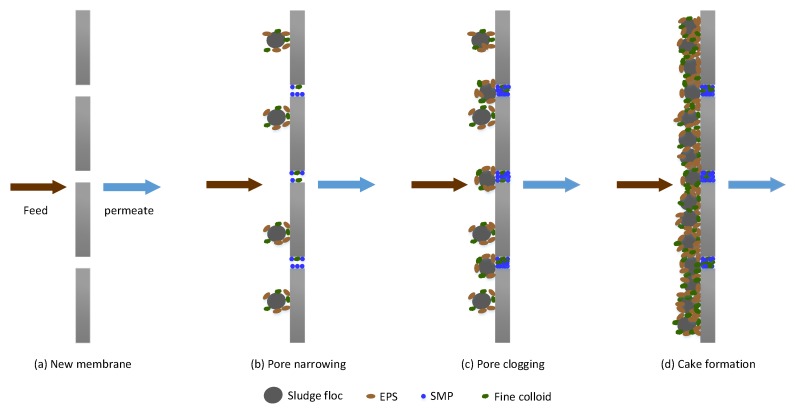
Mechanisms of membrane fouling in membrane bioreactors (MBR).

Fouling in MBRs occurs in different forms, namely, pore narrowing, pore clogging and, cake formation. Pore clogging refers to the blocking of membrane micro pores by foulants. Pore clogging depends, to a large extent, on the size of the particle and the membrane pore size. The attachment of the materials in the pores is aided by sticky substances in the solution. Cake formation, on the other hand, results from the continuous accumulation of bacteria clusters, biopolymers and inorganic matter, which form a layer (biocake) on the membrane [22]. The cake layer increases membrane filtration resistance. Membrane fouling mechanisms in MBRs are schematically illustrated in Figure 1.

In operational terms, membrane fouling decreases the permeate flux when the MBR is operated at constant transmembrane pressure (TMP), and results in the increase of TMP when the MBR is operated at constant permeate flux. At constant flux operation, a sharp increase in TMP indicates severe membrane fouling. This sudden TMP increase is called a “TMP jump”. TMP jump has been described as a three-stage process [1,23]: Stage 1—an initial “conditioning” fouling, which is caused by initial pore blocking and solutes adsorption; stage 2—linear or weakly exponential gradual rise in TMP due to biofilm formation and further membrane pore blocking; and stage 3—a sudden rapid increase in the rate of TMP rise (dTMP/dt) [3]. Stage 3 is thought to be the consequence of severe membrane fouling, and is believed to be due to successive closure of pores and changes to the local flux resulting from fouling, which causes local fluxes to exceed the critical value, hence, acceleration of particle deposition [24,25] and sudden changes of the cake layer structure [23]. Bacteria in the inner biofilms tend to die due to oxygen limitations, thereby releasing more EPS [26]. Once stage 3 occurs, membrane cleaning is required. The practical implication of this is that a delay in stage 3 will allow for a reduction in membrane cleaning frequency, which will ultimately result in MBR operational cost savings. Thus, one key objective of fouling control is to retard TMP jump through modification of sludge characteristics (MLSS, floc size, EPS content, and apparent viscosity) or lowering of operational flux [2].

### 2.1. Classification of Foulants

Membrane foulants in MBR can be grouped into biofoulants, organic foulants and inorganic foulants based on their biological and chemical characteristics [27].

#### 2.1.1. Biofoulants

Biofoulants refer to the bacteria or flocs whose deposition, growth and metabolism on the membrane results in fouling [2]. For a start, one bacteria cell may attach to the membrane surface or inside its pores and, after some time, the cell multiplies into a cluster of cells, leading to the formation of biocake, and hence reduced permeability. The bacteria (biofoulants) and their metabolic products contribute to fouling [28]. Essentially, membrane biofouling is a two-step process, starting with early bacterial attachment, followed by multiplication of bacteria on the membrane surface [29]. Some publications expand the definition of biofoulants to include the metabolic products of these clusters of bacteria cells [28]. However, in this paper, the organic substances produced by microorganisms are considered organic foulants (Section 2.1.2.) for the purpose of research on their mitigation strategies. Membrane biofouling is one of the most important operational problems in membrane-based systems [30].

#### 2.1.2. Organic Foulants

Organic foulants in MBRs refer to biopolymers, e.g., polysaccharides and proteins, of which deposition on the membrane results in a decline of membrane permeability. These foulants are found in metabolic products of bacteria, which are collectively called EPS. Compared to large particles, such as sludge floc, the deposition of organic foulants on the membrane surface is more difficult to remove [5]. In an experiment to investigate membrane fouling under various operational conditions using a laboratory-scale submerged MBR with a hollow-fibre membrane module, Wang and Li [31] reported that biopolymers are important foulants and have a significant impact on membrane fouling. Findings from their experiment further revealed that the rate of membrane fouling in the reactor correlated with the biopolymer concentration in the sludge suspension under different conditions.

In addition to EPS, research has indicated that MBR sludge also contains a pool of large-sized free organic solutes called biopolymer clusters (BPC) [32]. BPCs result from the clustering of loose EPS and soluble microbial products (SMPs) in the sludge cake [33]. BPCs are much bigger than SMPs and are composed primarily of biopolymers and with few microorganisms [33,34]. The few microorganisms in BPCs make them different from bacterial flocs. Due to the bigger size of BPCs, they are retained by the membrane in MBRs and are thus, not found in the MBR effluent. The large membrane surface in an MBR provides a conducive environment for BPC formation and growth; and, the formation and accumulation of BPCs in MBR causes serious membrane fouling [34]. In an experiment to investigate the fouling propensity of MBR sludge, Sun *et al.* [35] reported that increasing the concentration of BPCs by 20% and 60% from about 3.5 mg/L in the mixed sludge liquor remarkably raised the rate of fouling by 120% and 300%, respectively. This indicates that BPCs in the MBR suspension have a significant effect on the fouling potential of the sludge. Research has also been conducted to find ways of mitigating membrane fouling due to BPCs. It has been reported that the ozonation of BPCs can reduce the negative role of BPCs in membrane fouling [35]. In this experiment, 0.03 mg O_3_/mg total organic carbon (TOC) of BPCs reduced the mean BPC size from 38 to 27 μm and increasing the dosage to 0.3 mg O_3_/mg TOC of BPCs further reduced the size to 12 μm. It was further reported that ozonation also modified BPCs surface properties, leading to an increase in the filterable fraction and a reduction in the viscosity of the mixed liquor.

#### 2.1.3. Inorganic Foulants

Inorganic foulants are a group of inorganic substances that precipitate onto the membrane surface or into the membrane pores, resulting in membrane fouling. Examples of such substances include cations and anions such as Ca^2+^, Mg^2+^, Fe^3+^, Al^3+^, SO_4_^2−^, PO_4_^3−^, CO_3_^2−^, OH^−^, *etc.* [36,37]. These species precipitate onto the membrane surface due to hydrolysis which leads to pH change, and oxidation [31]. Essentially, inorganic fouling is produced from the chemical precipitation of inorganic species and/or biological precipitation of inorganic-organic complexes [36]. While moderate amounts of metal ions, such as Ca^2+^ (up to 280 mg/L), can be beneficial in controlling and improving biofouling due to binding and bridging EPS (hence, enhanced bioflocculation), high concentrations (above 800 mg/L) have been shown to significantly increase inorganic fouling due to high inorganic precipitate content of the MBR mixed liquor [38]. Inorganic fouling is also termed “mineral scale” in order to differentiate it from biofouling and organic fouling [39]. Crystallization and particulate fouling are the two key mechanisms that play critical roles during inorganic membrane fouling in MBR. In crystallization, precipitation of ions is the pathway to deposition at the membrane surface; while particulate fouling is the deposition following convective transportation of colloidal particulate matter in the solution to the membrane surface [40]. To remove inorganic precipitation from the membrane surface, chemical cleaning is usually the adopted procedure, as it is more effective than physical cleaning [2].

## 3. Factors Affecting Membrane Fouling in MBR

**Figure 2:**
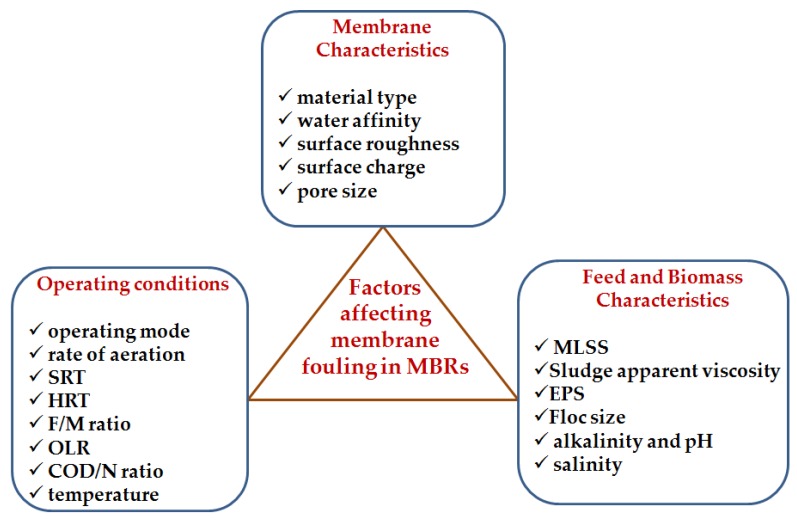
Factors affecting membrane fouling in MBRs.

Various factors affect membrane fouling in MBRs. These factors can be grouped into three categories, namely: membrane characteristics, operating conditions, and feed and biomass characteristics. Figure 2, below, is an illustration of the different factors, followed by their discussion in the subsections that follow.

### 3.1. Membrane Characteristics

#### 3.1.1. Membrane Material

The material the membrane is made of has an impact on its fouling propensity in MBRs. Based on the membrane material, membranes can be classified into: ceramic membranes, polymeric membranes, and composite membranes. Ceramic membranes exhibit good filtration performance due to their high chemical resistance, integrity, inert nature and ease of cleaning leading to low operating costs [13,41,42]. Ceramic membranes are also highly hydrophilic [5], which makes them more fouling resistant. However, their high cost of fabrication and fragile nature [41] make them economically unfeasible for use in MBR applications. Polymeric membranes are the most common membrane types available. Polymeric membranes have good physical and chemical resistance but are mostly hydrophobic [5]. Examples of polymeric membrane materials include polyvinylidene fluoride (PVDF), polyethersulfone (PES), polyacrylonitrile (PAN), polysulfone (PS), polyethylene (PE), polyvinyl butyral (PVB), cellulose acetate (CA), polypropylene (PP), polytetrafluoroethylene (PTFE), *etc*. Due to their hydrophobic nature, polymeric membranes tend to foul easily, but they tend to be widely used now due to the ease of fabrication of the pore sizes. Composite membranes are membranes produced from two or more materials to combine the strengths of the constituent materials in the final product. Typically, one material constitutes the active surface and another forms the support layer [13]. In composite membrane applications, hydrophobic membranes are coated with hydrophilic polymer to overcome the fouling shortcoming.

The implication of this is that membrane properties can be modified as a fouling abatement measure. Hence, recent research has focused on modifying membrane materials to mitigate fouling in MBRs. Yu *et al.* [43] modified a PP membrane surface using air plasma treatment, and used the modified membrane to investigate the antifouling characteristics in a submerged MBR. The findings showed that flux recovery following water and caustic cleaning was 35% higher than that of the unmodified membrane [43]. Another investigation has been conducted on the modification of PES ultrafiltration (UF) membranes using different forms of nanosilver [44]. Findings from this research revealed that the modified membranes fouled less than the control. Another innovative membrane modification for fouling mitigation is the production of photocatalytic nanocomposite membranes. In this regard, Moghadam *et al.* [45] evaluated the flux performances and antifouling properties of PVDF-TiO_2_ nanocomposite membranes, with and without UV irradiation. The authors found that the composite membranes (compared to unmodified PVDF membranes) reached flux stabilisation very rapidly both in darkness and under UV conditions; in addition, the PVDF-TiO_2_ nanocomposite membranes presented the best flux recovery ratio when filtration was combined with UV irradiation at 365 nm. Lower membrane cleaning frequency was also reported for the PVDF-TiO_2_ nanocomposite membrane compared to the unmodified PVDF membrane.

However, the long term effects of the modified membranes need further investigation especially for membranes modified with nano-particles as these nano-particles can have human health and ecotoxicological impacts [46].

#### 3.1.2. Water Affinity

The water affinity (hydrophilicity or hydrophobicity) property of the membrane material affects fouling in MBRs. The water affinity behaviour of a membrane material is determined by measuring the contact angle of a water drop on its surface [47]. Smaller angles indicate hydrophilicity, whereas larger angles indicate hydrophobicity. Due to the hydrophobic interactions occurring between the membrane material, microbial cell and solutes, membrane fouling is more severe in hydrophobic membranes compared to hydrophilic membranes [1]. This is because the more hydrophilic a membrane material is, the less the adsorption of macrosolutes in the wastewater, such as proteins, there are. Hydrophobic materials, on the other hand, tend to adsorb hydrophobic substances in the wastewater, resulting in fouling. To strike a balance, composite membranes are produced by coating hydrophobic membranes with a thin layer of hydrophilic material to combine the sturdiness of the former and the low fouling propensity of the latter [47].

#### 3.1.3. Membrane Surface Roughness

The surface roughness of the membrane material also has some influence on membrane fouling in MBRs. Membranes with homogeneous surfaces are less subject to be fouled than those with uneven surfaces [47]. Research findings have indicated that membranes with higher surface roughness foul faster [48]. This is because the rough membrane surface provides a valley for the colloidal particles in the wastewater to accumulate on [49], leading to the blocking of the valleys thus increasing the severity of fouling for rougher membrane surfaces [50]. However, a study conducted to investigate the relationship between surface roughness and membrane fouling in MBRs found that membranes having higher projections on their outer surface exhibited a higher antifouling property, with the recovery of permeability after backwashing following the same trend [51]. This is attributable to the accumulation of foulants in the valley between the projections. Therefore, although rough surfaces increase the propensity for fouling, rougher surfaces with protruding projections may trap the foulants in their valleys whilst still functioning normally.

#### 3.1.4. Membrane Surface Charge

The membrane surface charge is another property of importance in relation to membrane fouling especially if there are charged particles in the feed. It has been indicated that most membrane materials are negatively charged under normal conditions [47]. This is partly attributable to the colloidal particles that deposit on the membrane surface [49]. Some cations in the MLSS like Ca^2+^ and Al^3+^ will react with the negatively charged membranes surfaces, leading to inorganic fouling.

#### 3.1.5. Membrane Pore Size

Generally, membranes used in wastewater treatment are broadly grouped into two: porous membranes and non-porous membranes. Porous membranes employ straining, sieving, or size exclusion to separate particles, e.g., microfiltration (MF), ultrafiltration (UF), and loose end nanofiltration (NF) membranes [39]. Non-porous membranes, on the other hand, make use of the differences in diffusivity or solubility between the solvent and the solute in the membranes for separation [52], as cited by Shirazi *et al.* [39]. Examples of non-porous membranes include tight end (NF) and reverse osmosis (RO) membranes. Because the separation mechanism required in MBRs is sieving (size exclusion), MF and UF membranes are typically used [5], thus allowing the complete physical retention of bacterial flocs and virtually all suspended solids within the bioreactor [1].

**Figure 3:**
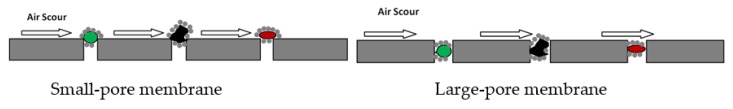
Schematic of pore blocking on small-pore and large-pore membranes.

The pore size of the membranes in relation to the sizes of the particles in the wastewater feed stream in MBRs can have an effect on membrane fouling. Pore blocking mechanism tends to increase with increasing membrane pore size [47]. This is because, it is easier for fine particles (smaller than the membrane pore size) to enter the membrane pores and get trapped in them, resulting in pore blocking [53]. With smaller pores, large particles rapidly form a top layer on the membrane and collect the smaller particles. The resulting layer formed on the membrane surface can be easily removed by air scouring or the turbulence resulting from cross-flow filtration. This is schematically illustrated in Figure 3 below. Generally, the effects of membrane pore size on fouling depend greatly on the composition of the feed stream, particularly the particle size distribution.

Miyoshi *et al.* [54] investigated the effect of different polymeric membrane materials on the relationship between membrane pore size and fouling in an MBR using cellulose acetate butyrate (CAB), PVB, and PVDF membranes. Their result revealed that membrane fouling decreased with increasing membrane pore size for PVDF membranes; but CAB membranes exhibited an opposite trend with smaller pores showing less fouling tendency than those with larger pores [54]. This indicates that the best membrane pore size to abate membrane fouling in MBR differs for different polymeric materials. Hence, MBR operating parameters ought to be carefully selected for different polymeric membrane materials.

### 3.2. Operating Conditions

#### 3.2.1. Operating Mode

Two operating modes are typically used in MBRs namely, constant TMP with variable permeate flux and constant permeate flux (L/m^2^ h) with variable TMP. The latter is the preferred mode in MBRs as it can readily handle fluctuations in influent hydraulic loading [47]. When operating at constant permeate flux, membrane fouling is observed by TMP jump. The critical flux needs to be determined in constant flux operation, because it is an important parameter in MBR operation [5]. The determination of critical flux is presented by Diez *et al.* [55]. The critical flux is a quantitative parameter for the filterability of different membranes and/or different activated sludge mixed liquors [56]. Critical flux is the flux above which the deposition of solids on the membrane surface becomes evident (cake or gel formation) [55]. In practical terms, however, critical flux is not attainable in MBR. The tendency, therefore, is to operate at the sustainable flux rather than the critical flux. Sustainable flux refers to the flux above which membrane fouling rate is economically and environmentally unsustainable [57] or the flux for which there is gradual increase in TMP at an acceptable rate, such that chemical cleaning is unnecessary [1]. In essence, the sustainable flux, which is less than the critical flux, provides an acceptable rise in TMP. This operational flux plays a key role in MBR fouling. For a given MBR system, operating at a flux below the critical flux prevents excessive deposition of biomass on the membrane surface [2,44,58].

#### 3.2.2. Rate of Aeration

Aeration plays a dual role in aerobic MBRs. It supplies oxygen for the biological processes and serves as a way of dislodging the cake layer formed on the membrane surface (air scouring). The oxygen supplied via aeration facilitates the biodegradability and cell synthesis of the biomass [12]. Research has shown that increasing the rate of aeration in an MBR leads to a reduction in membrane fouling [59,60]. In a study to investigate the impact of aeration velocity on membrane fouling in a pilot-scale submerged MBR, Yigit *et al.* [61] reported that increase in aeration velocity positively impacted fouling control. However, the degree of such a positive effect was substantially reduced as MLSS increased. This is attributable to increased viscosity resulting from elevated levels of MLSS. Air scouring rates in the literature range from 3 L air/min m^2^ to 12 L air/min m^2^ [17].

While a high rate of aeration can minimise membrane fouling through the scouring action, it also has an impact on biomass characteristics. High aeration intensities have been associated with the breakage of sludge flocs and subsequent production of SMPs [2]. Higher aeration rates also increase the energy consumption leading to an increase in operating costs [62,63]. Thus, an optimum aeration intensity needs to be found which strikes a balance between these. It has been indicated that going beyond the critical aeration intensity increases membrane fouling as the shear breaks up large flocs [64]. In this regards, Nywening and Zhou [63] studied the effectiveness of membrane fouling and scouring aeration in three pilot-scale submerged MBRs operated at a series of permeate fluxes, scouring aeration intensities and cyclic aeration frequencies to treat municipal wastewater. They developed a very useful empirical relationship relating the stable reversible fouling resistance to sustainable permeate fluxes and scouring aeration intensities, which is shown in Equation (1).

(1) $$R_{f\left(i\right)\;}=R_{O\left(ref\right)}\left[\;\;\;\frac{-1}{\left(\;\frac{Jc}{J_{O\left(ref\right)}}-1\right)}\left(\frac{J_{i}}{J_{O\left(ref\right)}}\;-1\right)+1\right]{\left(\frac{Q_{\left(i\right)}}{Q_{O\left(ref\right)}}\right)}^{l}$$

where:

*R_f_*_(*i*)_ is the fouling resistance at operating conditions *i*, m^−1^;
*R_O_*_(*ref*)_ is the fouling resistance at reference point *O*, m^−1^;
*J_C_* is the critical flux, L/m^2^ h;
*J_O_*_(*ref*)_ is the reference permeate flux at point *O*, L/m^2^ h;
*J_i_* is the permeate flux at operating condition *i*, L/m^2^ h;
*Q*_(*i*)_ is the scouring aeration intensity at operating condition *i*, m^3^/h;
*Q_O_*_(*ref*)_ is the scouring aeration intensity at reference operating condition *O*, m^3^/h;
*l* is the scouring aeration intensity exponent, which is approximately −2.

#### 3.2.3. Solids Retention Time (SRT)

The SRT is a very important factor affecting membrane fouling in MBRs [13]. The SRT varies with the formation of EPS. A majority of studies indicate that increasing the SRT results in a decrease in the concentration of EPS as the biomass stays longer in the system, and lowering the SRT increases the amount of EPS [65,66,67,68]. High SRTs produce starvation conditions in the bioreactor creating an enabling environment for reduced formation of EPS, low sludge production and nitrification [5,69]. However, extremely high SRTs are not desirable as they rather increase membrane fouling due to the accumulation of matters (high MLSS accumulation) and increasing sludge viscosity [66]. Excessively high SRTs can also lead to high biomass concentrations resulting in reduced aeration efficiency [53]. In an experiment to investigate the effect of SRT on membrane fouling in MBR, Van den Broeck *et al.* [53] reported lower membrane fouling rates at 30 days and 50 days SRTs compared to 10 days SRT. It has been indicated that operating above 50 days tends to increase fouling [13].

Similarly, operating at very low SRTs (down to two days) significantly increases membrane fouling [1]. This has been attributed to increased EPS (bound and soluble) concentration at these low SRTs [2]. Extremely low SRTs also result in a reduction in MBR performance as a result of low biomass concentration [70].

#### 3.2.4. Hydraulic Retention Time (HRT)

The HRT has indirect effect on membrane fouling as it determines, with other operating parameters, the sludge characteristics. Most researchers agree that as HRT decreases, the rate of membrane fouling in MBRs increases [71,72] due to an increase in sludge viscosity and EPS concentrations [2]. The decrease in HRT stimulates the release of EPS from bacterial cells, causes overgrowth of filamentous bacteria, and the formation of irregular large flocs. Additionally, the decrease in HRT causes an increase in MLSS concentration and sludge viscosity which are predominant factors that affect hydrodynamic conditions of MBR systems [30,70]. Isma *et al.* [73] investigated the influence of HRT and SRT on membrane fouling using synthetic wastewater. Their study, which used SRTs of 4, 15 and 30 days at HRTs of 4, 8 and 12 h, respectively, found that lower membrane fouling and slower rise in TMP were observed at the longest SRT (30 days) and longest HRT (12 h). Similarly, a study of the effect of microbial activity and fouling potential in submerged anaerobic MBRs operated at HRTs of 14, 16, and 20 days found that decreasing the HRT from 20 to 14 days resulted in increased EPS (bound EPS and SMPs) production, and hence severe fouling [74].

#### 3.2.5. Food–Microorganisms (F/M) Ratio

The F/M ratio is an important operational parameter in biological wastewater treatment systems. In order to determine the influence of F/M ratio on membrane fouling, Kimura *et al.* [19] investigated fouling in three identical pilot-scale MBRs using real municipal wastewater under different operating conditions. Their results indicated that the F/M ratio influences the nature of the foulants as high F/M correlated with higher proteinaceous foulant [19]. Similarly, Trussell *et al.* [75] reported that the rate of membrane fouling in MBRs increases with increasing F/M ratios. High values of the F/M ratio can also elevate EPS levels due to high food utilisation by biomass [70]. Another study also reported that decreasing F/M ratios resulted in a reduction in the concentration of EPS [76]. It is therefore desirable to operate at lower F/M ratio values.

#### 3.2.6. Organic Loading Rate (OLR)

The OLR is one of the most important parameters affecting the operation of biological wastewater treatment systems [77]. In MBR, Zhang *et al.* [78] investigated the effect of constant and variable influent OLR on membrane fouling using two identical laboratory-scale submerged MBRs operated for 162 days at an SRT of 30 days; the influent OLR was kept constant in one MBR and varied in the other. They reported that membrane fouling during start-up period in the MBR fed with variable OLR was more significant than the MBR fed with constant OLR. However, at the MBR stable state (when the MBR systems gradually stabilised in terms of biomass concentration and TOC removal), the fouling tendency was clearly reversed with less membrane fouling observed for variable feed OLR. In another study, Johir *et al.* [79] investigated the effect of OLR on membrane fouling in MBRs operated at six different OLRs ranging from 0.5 to 3.0 kg COD/m^3^ day (COD = chemical oxygen demand) at constant HRT and SRT of 8 h and 40 days, respectively. Findings from their study revealed that higher fouling rates were observed at higher OLRs (2.75–3.0 kg COD/m^3^ day) with more hydrophilic substances at higher OLRs.

#### 3.2.7. Chemical Oxygen Demand/Nitrogen (COD/N) Ratio

The COD/N ratio is one of the most important parameters for the growth of microorganisms. It also plays a key role in nutrients’ removal (particularly nitrification and denitrification) [80]. This operating parameter has also been reported to correlate well with membrane fouling in MBRs. Feng *et al.* [81] studied the effect of COD/N ratio on membrane fouling in two identical submerged MBRs operated in parallel at COD/N ratios of 10:1 and 5:1, respectively. They reported that operation at COD/N ratio of 10:1 remarkably reduced membrane fouling rate through the slower rise in TMP compared to the MBR operated at COD/N ratios of 5:1. They further found that the duration of stage 2 of the TMP jump was twice (over 30 days) as long in the COD/N ratio of 10:1 compared 5:1. Similarly, Hao *et al.* [82] investigated the effect of COD/N ratio on membrane fouling in MBRs operated at three different ratios (100:5, 100:2.5, and 100:1.8) for over a year. Findings from their research showed that increasing the COD:N ratio from 100:5 to 100:1.8 resulted in an improved membrane performance and a longer operation period before membrane cleaning; and, the bound EPS/SMP composition, such as protein to carbohydrate ratio, played a more significant role in controlling fouling than the total amount of bound EPS and SMP produced.

However, there is inconsistency on the effect of COD/N ratio on membrane fouling in the literature. Han *et al.* [83] compared membrane filtration performance in anoxic/oxic MBRs with COD/N ratios of 9.9 and 5.5. They reported more membrane fouling at high COD/N ratio as the increase in COD/N ratio led to an elevated production of humic acids in SMPs and carbohydrates, proteins, and humic acids in LB-EPS. Similarly, Gasmi *et al.* [84] found lower specific resistance to filtration and membrane fouling rate at low COD/N ratios (2.3 and 1.5). Yang *et al.* [85] also reported that low COD/N ratio of 3.5 greatly facilitated membrane fouling control by a simple aeration and backflushing strategy.

#### 3.2.8. Temperature

Temperature is known to influence the rate of biodegradation. In MBRs, temperature impacts membrane fouling by altering the MLSS characteristics. It has been reported that decreasing operational temperature causes the bacteria to release more EPS [86]. Very low temperatures are associated with an increased occurrence of filamentous bacteria, which produce more SMPs in the MLSS [30], hence more propensity for membrane fouling. Deflocculation, diffusitivity, biodegradation, and adsorption in the MBR have also been indicated to depend on temperature [3]. Generally, four phenomena have been put forward to explain the increased membrane fouling at lower temperatures in MBRs: (a) increased viscosity which reduces the shear stress generated by aeration; (b) intensified deflocculation which reduces the size of biomass floc and releases EPS and submicron particles into the MLSS; (c) reduced back transport velocity; and (d) lower biodegradation of organic matter (COD) [87]. Ma *et al.* [86] investigated the effect of temperature on membrane fouling in a pilot-scale submerged MBR and reported much higher concentration of EPS (bound EPS and SMPs) under low temperature, with SMPs decreasing from 28.1 mg/g-MLSS at 8.7 °C, to 2.2 mg/g-MLSS at 19.7 °C with a difference of 25.9 mg/g-MLSS. Furthermore, sudden changes in temperature have been reported to cause spontaneous release of SMPs and increased fouling rates [88]. Deflocculation of sludge flocs have been reported to occur following a temperature increase from 30 °C to 45 °C which led to turbidity increase and increase in SMPs concentration [89]. Sudden temperature increase has also been indicated to deteriorate biomass and decrease proteins in EPS [30]. To overcome these problems, it is suggested to operate MBRs at ambient temperature and avoid sudden temperature changes (increase or decrease). If low temperatures are inevitable, aeration needs to be intensified to overcome the increased viscosity.

### 3.3. Feed and Biomass Characteristics

The feed (wastewater) and biomass characteristics play key roles in membrane fouling in MBRs. The complex interactions amongst the constituents of biomass and the membrane material affect membrane fouling in MBRs.

#### 3.3.1. Mixed Liquor Suspended Solids (MLSS)

The MLSS contains bacteria flocs, EPS, colloids, microsolutes and macrosolutes. MBRs are operated at MLSS concentrations much higher than ASP. However, the higher concentration of MLSS in MBRs accelerates membrane fouling as a result of high suspended solids [90]. Research findings have indicated that membrane permeability decreases with increasing MLSS concentrations [60]. Wu and Huang [91] reported that operating at MLSS concentrations above 10,000 mg/L greatly increased the viscosity, which, in turn, affected the filterability. Yigit *et al.* [61] studied the impact of operational conditions and biomass characteristics on membrane fouling in a submerged MBR and reported that the concentrations of both polysaccharides and proteins fractions of EPS increase with increasing MLSS concentration. The study further found that the increase in MLSS resulted in a significant decrease in membrane permeability and increased fouling rate at each flux tested. These findings indicate that membrane fouling increases with increasing MLSS concentrations. Similarly, if there is a dominance of filamentous bacteria in the MLSS, there is a tendency for filamentous bulking to occur. Filamentous bulking can significantly increase the production of SMPs which in turn substantially increase the fouling of membranes [68].

However, reports about the effects of MLSS on membrane fouling in MBRs are not consistent in the literature. For example, Rosenberger *et al.* [92] reported reduced membrane fouling as MLSS concentration increased until 15 g/L when the trend reversed and the fouling rate increased at concentrations above 15 g/L. This can be explained in terms of remarkable changes to the sludge rheology. Other studies report no (or little) effect of MLSS concentration on membrane fouling in MBR including Rosenberger *et al.* [93] (MLSS = 9–14 g/L: no effect), Le-Clech *et al.* [94] (MLSS = 4.4–11.6 g/L: No impact between 4 and 8 g/L, but slightly less fouling at 12 g/L), and Brookes *et al.* [95] (MLSS = 6–18: Similar rate of fouling for flux below 10 L/m^2^ h and slightly lower fouling rates for higher fluxes). Thus, there is no clear correlation between MLSS concentration and membrane fouling.

#### 3.3.2. Sludge Apparent Viscosity

Viscosity is the measure of a fluid’s resistance to gradual deformation by shear or tensile stress. Since MBRs are operated at high MLSS, the total suspended solids content is also very high and this results in higher viscosity values [96]. The high viscosity in MBRs can limit oxygen transfer leading to higher energy consumption for aeration. Trussell *et al.* [60] reported that increasing the viscosity results in membrane permeability decline. Typically, there is a critical MLSS concentration below which sludge viscosity stays low and increases only slowly with increasing concentration [1]. Operating above the critical MLSS concentration results in viscosity increasing exponentially with MLSS concentration [97]. Depending on the operating conditions, the critical MLSS value has been observed to range from 10,000 to 17,000 mg MLSS/L [1]. With higher MLSS viscosity, there is increased rate of membrane fouling in MBR.

#### 3.3.3. Extracellular Polymeric Substances (EPS)

Extracellular polymeric substances (EPS) are waste products of bacteria originating from microbial metabolites, cell lysis, or unmetabolised wastewater components [3]. EPS are known to significantly affect the physico-chemical properties of microbial aggregates such as surface charge, structure, settling properties, flocculation, adsorption ability, *etc.* [98]. They primarily comprise proteins, polysaccharides (carbohydrates), humic acids, nucleic acids, lipids and uronic acids [68,99,100]. Proteins and polysaccharides are the major components of EPS [98] and are the components typically measured as an indication of the amount of EPS. In terms of fouling, proteins exhibit hydrophobic properties while polysaccharides are hydrophilic in nature and this implies that the polysaccharides fractions of EPS have a higher propensity for fouling than protein fractions [61,101], given that hydrophilic membranes are used. EPS are divided into two, namely bound EPS and soluble EPS (also referred to as SMPs) [100]. Bound EPS are further split into loosely bound EPS (LB-EPS) and tightly bound EPS (TB-EPS) [102]. SMPs and bound EPS are considered the major foulants in MBRs [2,36,103,104] as they have multiple interactions with all other foulants [100]. The aggregation of biomass can happen by participation of bound EPS and SMPs, which provides a highly hydrated gel matrix [12,104]. These substances act as a “glue” to keep the microbial aggregates together.

Bound EPS are characterised by adhesion to sludge flocs [99,100]. They are located at or outside the cell surface. Bound EPS facilitate the aggregation of biomass to form microbial aggregates, which alters the flocculation ability, surface charge, hydrophobicity, and sludge viscosity [13]. This alteration affects membrane fouling. TB-EPS provide the inner layer, while LB-EPS are distributed on outer surface of the microbial aggregates. Bound EPS are construction materials for microbial aggregates like sludge flocs and biofilms. In these microbial aggregates, EPS have many charged groups, such as hydroxyl, carboxyl, sulfhydryl, phosphoric and phenolic groups; and polar groups, such as hydrophobic regions in carbohydrates, aliphatics in proteins and aromatics [30]. The presence of both hydrophobic and hydrophilic groups in EPS implies that they are amphoteric in nature. Since the hydrophilic fraction is known to foul the membrane (hydrophilic membrane) more than the hydrophobic fraction, the proportion of hydrophobic to hydrophilic organics plays an important role in the formation of fouling [105]. Thus, the ratio of proteins (hydrophobic) to polysaccharides (hydrophilic) in EPS governs membrane fouling in MBRs, especially cake layer formation [12].

SMPs are the organic compounds released into solution from substrate metabolism and biomass decay [5,106]. SMPs released from substrate metabolism are referred to as substrate utilisation-associated products (UAP) and those resulting from biomass decay are termed biomass-associated products (BAP) [107]. SMPs composition is dominated by polysaccharides, proteins and humic acids [68]. It has been reported that SMPs contribute more to membrane fouling than colloids in MBRs [91]. Another study found that MLSS and bound EPS are not major contributors to fouling when compared to SMPs [99]. The effect of SMPs on membrane fouling in MBRs depends on their concentration, membrane materials, and mode of operation [13]. This implies that the control of SMPs concentration in MBRs is a key factor in controlling fouling. Research indicates that SMPs concentration decreases with increasing SRT, and higher SMPs concentrations are observed at lower dissolved oxygen (DO) concentrations [2]. In contrast to bound EPS, SMPs more easily penetrate into sludge flocs spaces and the membrane pores, and hence there is more fouling.

#### 3.3.4. Floc Size

Microorganisms tend to aggregate and form flocs in biological wastewater treatment systems. The size of the flocs formed aids the liquid-solids separation of the treated water from the MLSS. In MBRs, a wide range of sizes for these flocs have been reported in the literature with size ranging from 5 to 240 μm [1,108]. In a recent study, Shen *et al.* [109] investigated the impact of floc size on membrane fouling in a submerged MBR treating synthetic wastewater. Findings from their study, which had sludge floc size above 1 μm, revealed that a decrease in floc size slightly increased the specific energy barrier but greatly increased the attractive specific interaction energy [109]. This implies an increase in adhesion ability of small flocs to the membrane surface, hence more fouling. The practical implication of this is that the larger the floc size, the better it is for fouling reduction. Current research has thus focused on increasing the floc size using aerobic granulation [11,110,111,112,113], activated carbon addition [114], or zeolite addition [115]. The increment in floc size improves filtration by reducing fouling.

#### 3.3.5. Alkalinity and pH

Alkalinity and pH are important parameters in biodegradation. In relation to MBR, these two factors affect the rate of membrane fouling. It has been reported that low pH values result in increased adsorption of MBR-originated EPS onto the membrane [116]. Another study found the highest EPS flocculation tendency at pH 4.8 [117]. Zhang *et al.* [118] also reported that a repulsive energy barrier exists between sludge flocs and the membrane surface; and, this repulsive energy barrier decreases with decreasing pH which in turn facilitates the attachment of foulants to the membrane. Similarly, Sanguanpak *et al.* [119] reported more severe fouling at low pH (5.5) due to more EPS formation in an MBR treating landfill leachate. All these studies agree that a decrease in the pH of the mixed liquor increases the rate of membrane fouling in MBR. Within the bioreactor, nitrification process generates acid which would lower the pH [120]. To keep the bioreactor at an optimum pH range, alkalinity is required in the feed to buffer the H^+^ generated in the nitrification process. If alkalinity is low in the feed, the deficiency would need to be accounted for by adding extra alkalinity.

It should be noted that since inorganic fouling results from chemical precipitation and biological precipitation, pH can affect chemical precipitation. It has been indicated that high pH (8–9) increases the precipitation of CaCO_3_ [2]. However, moderate amounts of calcium precipitates can be beneficial in controlling biofouling due to binding and bridging EPS (hence, enhanced bioflocculation). Arabi and Nakhla [38] reported that calcium concentration of 280 mg/L improved membrane permeability, while higher concentrations of 830 mg/L resulted in significant membrane inorganic fouling.

#### 3.3.6. Salinity

Salinity is known to have adverse effects on biological systems. In MBRs, it has been demonstrated that the presence of salts in the MLSS cause chemical precipitation and electrostatic attraction towards the surface of the membrane [48]. High salinity also modifies biomass characteristics in the system. Reid *et al.* [121] studied the impact of high salinity (up to 5000 mg/L) on activated sludge characteristics and membrane permeability in a submerged MBR. Findings from their research revealed that high salinity greatly affected the physical and biochemical properties of activated sludge by increasing bound EPS and SMPs concentrations, and decreasing membrane permeability (increasing membrane fouling) [121]. Similarly, Jang *et al.* [122] investigated the effects of salinity on membrane filtration in MBRs with high salt loadings and reported that high salt concentrations accelerated membrane fouling through increased pore blockings. This implies high salinity altered biomass characteristics like EPS, floc size and zeta potential, which eventually resulted in increased membrane fouling. In another study, Di Bella *et al.* [123] reported that the MBR plant exhibited high biomass respiratory activity and high removal efficiencies at normal salinity content. However, as salinity increased, a decrease of respiration rates was observed alongside increased fouling (cake deposition) attributable to the deterioration of high EPS concentration.

In addition, the ionic composition of the wastewater also plays a role in floc formation. It has been indicated that floc structure and strength strongly depend on the ionic composition and concentration [124]. High concentrations of multivalent cations, e.g., Mg^2+^ and Ca^2+^, are known to form strong and compact flocs [125,126]. This can be explained by the divalent bridging model where Ca^2+^ and other divalent ions bridge the EPS negatively charged sites, thereby forming a matrix of EPS and single cells. Monovalent cations, on the otherhand, lower floc strength rather [127]. Hence, the presence of polyvalent cations in relation to monovalent cations, in spite of high salinity, can aid the formation of strong bioflocs which would aid membrane filtration.

A summary of the different factors affecting membrane fouling in MBRs and their respective effects are presented in Table 1 below.

## 4. Current Research Trends for Membrane Fouling Abatement in MBR

### 4.1. Coagulant Addition

The addition of coagulants to water and wastewater treatment systems facilitates the formation of large flocs from fine particulates in solution. In MBRs, the coagulants help the formation of larger size sludge flocs which enhance membrane filtration. Alum [Al_2_(SO_4_)_3_] and ferric chloride (FeCl_3_) were reported to enhance filterability of MBR mixed liquor and ultimately controlled membrane fouling [128,129]. Wu *et al.* [130] studied the effects of polymeric coagulants on membrane fouling in MBRs using three coagulants: polymeric ferric sulphate (PFS), polymeric aluminium chloride (PACl), and polymeric aluminium ferric chloride (PAFC). They reported that the addition of these coagulants resulted in membrane fouling control through the reduction of the initial TMP and the rate of TMP increase. This was attributed to the ability of the coagulants to restrain the formation of gel layer, decelerate foulants development, and remove stable foulants from the surface of the membrane. PFS was found to be the most effective in controlling membrane fouling with an optimum dose of 1.05 mM Fe. The reduction of initial TMP and delayed rate of TMP increase when coagulants were added can be explained in terms of charge neutralisation and bridging of the SMPs. Coagulation could also minimise membrane fouling due to the flocculation of the particulates (colloids) in the MBR reactor brought about by coagulant addition. Wu *et al.* [131] further investigated the effect of PFS on membrane fouling characteristics and performance in a long-term (60 days) MBR operation. Findings from their study indicated that the addition of PFS efficiently impeded membrane fouling in long-term MBR operation; and, the optimum PFS dosage and dosing interval were 1.0 mM Fe and 15 to 30 days at an MLSS 7–10 g/L. Additionally, PFS increased the flocs size through the supply of positive charges for organic particles, hence enhanced charge neutralization.

In another study, Zhang *et al.* [132] investigated the ability of FeCl_3_ to retard membrane fouling in MBR. They reported that an optimum dose of 1.2 mM Fe(III) remarkably improved the filterability of the MBR mixed liquor; which, they attributed to the fact that Fe(III) supplied positive charges for soluble macromolecular substances and sludge flocs, and enhanced the function of charge neutralisation. The added Fe(III) interacted with the negatively charged EPS groups and enhanced the bioflocculation of small particles.

Furthermore, Mishima and Nakajima [133] investigated the mitigation of membrane fouling in MBR by coagulant addition in laboratory-scale batch and MBR experiments. They reported that ferric chloride performed slightly better than aluminium sulphate. Thus, using ferric chloride in the MBR process, they reported that during the 40-day MBR experiment, the reference MBR tank (without coagulant addition) was cleaned 18 times; the tank with 2.26 g/L coagulant addition was cleaned nine times; and the MBR tank with 4.52 g/L addition was cleaned only five times [133]. This indicates that, with coagulant addition, the fouling rate reduced appreciably. It has also been found that the addition of a very low-dose of green bioflocculant could achieve significant membrane fouling mitigation after 70 days of operation (TMP development of 2.5 kPa only) with less backwash frequency [134].

However, the addition of coagulants to the MBR mixed liquor can decrease the pH. The decrease in pH may affect the bioactivities of the MBR mixed liquor. In addition, coagulant overdosage can result in the deposition of the excess coagulant on the membrane surface. Research is, thus, needed on finding sustainable dosages that mitigate membrane fouling without lowering the pH.

### 4.2. Adsorbent Addition

Adsorbents provide a large surface area for the adsorption of materials in water and wastewater. In MBR, adsorbents offer the potential to adsorb dissolved organic polymers, notably SMPs, hence reducing membrane fouling propensity. Powdered activated carbon (PAC) is typically applied in MBRs for the purpose of reducing organic fouling and biofouling. PAC also serves as media for bacterial attachment and subsequent growth [5], hence, reducing their attachment onto the membrane surface and pores.

Ying and Ping [135] studied the effect of PAC dosage on membrane fouling using PAC dosages of 0, 0.75, and 1.5 g/L of wastewater, respectively. They reported that PAC application was effective in reducing the amount of EPS inside the microbial floc at PAC dosage of 0.75 g/L; and, the addition of PAC decreased the EPS deposited on the membrane. The cake resistance decreased as the PAC dosage increased. The optimum PAC dosage for organics removal and filtration flux was found to be 0.75 g/L. Similarly, Satyawali and Balakrishnan [136] studied the effect of PAC addition on sludge properties in MBR treating high-strength wastewater from an alcohol distillery in long-term operation (over 180 days). They reported that PAC addition decreased the SVI (sludge volume index) which improved sludge dewaterability. They further found that PAC addition did not substantially affect total EPS concentration; however, the composition of the SMPs in terms of proteins/polysaccharides ratio was altered resulting in a high proteins/polysaccharides ratio.

In another study, Remy *et al.* [114] investigated the effect of PAC on membrane fouling in two pilot-scale MBRs treating municipal wastewater. Findings from their research revealed that low PAC dosage (500 mg/L of sludge) combined with a relatively long SRT (50 days) resulted in about 10% improvement of the critical flux and a strong filtration period increment without significant fouling at high fluxes (50–70 L/m^2^ h). Other positives reported from their research include: Easier removal of gels deposited on the membrane at high fluxes; a reduction in the deposition of gel on the membrane surface after a long period of filtration; and a slight increase in the permeate quality. Torretta *et al.* [137] investigated the optimum dose of PAC in an MBR pilot plant by using low PAC doses: 0, 2, 5, 10 and 20 mg/L. Findings from this study showed that PAC addition was effective at the low doses (2 and 5 mg/L) by reducing the permeate flux loss (from 16% up to 27%, respectively). Rezaei and Mehrnia [116] found that the addition of the zeolite (clinoptilolite) resulted in significant improvement of sludge properties including 22.5% increase in MLSS, more accumulation of large particles (7%), 50% reduction in SMPs, and 66% reduction in TMP. The increase in flocs size, reduction in SMPs and TMP translate to remarkable membrane fouling abatement. Similarly, another study found that MBR with PAC in the mixed liquor exhibited low fouling tendency and prolonged filtration as compared to conventional MBR [138]. The authors further indicated that PAC stabilised the biomass in form of biological activated carbon with porous cake structure that prolonged filtration.

It has been indicated that higher concentration of fresh PAC in submerged MBR would offer enhanced simultaneous adsorption and biodegradation effects for the reduction of EPS, fine colloids and planktonic cells in MBR mixed liquor supernatant [139]. Membrane fouling abatement in MBRs through the addition of PAC has been attributed to the combined action of flocculation and adsorption [138]. The adsorption opportunity provided by PAC in MBRs is expected to enhance organics removal. In general, PAC addition to MBR acts as mobile carriers for active biomass, reduces membrane cake layer formation, and retains microorganisms by making the MBR both attached growth and suspended growth [140]. An additional advantage offered by enhancing MBRs with adsorbents (particularly PAC) is the removal of recalcitrant pollutants from wastewater [141].

Furthermore, Deng *et al.* [142] reported that the addition of sponge to the MBR resulted in lower biomass growth, less filamentous bacteria, reduced sludge viscosity, larger sludge flocs, and lower concentration of EPS and SMPs, leading to lower pore blocking resistance and lower cake formation when compared to conventional MBR [142]. This clearly shows that sponge addition can significantly alleviate membrane fouling in MBRs.

While the addition of adsorbents mitigates membrane fouling in MBR, further research is needed to establish optimum dosages of the various adsorbents. Operating above the optimum dosage can be counterproductive as it may rather increase sludge apparent viscosity, aggravate fouling through deflocculation, reduce mass transfer and sludge dewaterability [141]. It has also been indicated that overdose of adsorbents (especially PAC) may increase membrane fouling due to their potential to become foulants themselves through cake layer formation over the membrane and/or by blocking the membrane pores [135,143]. It is necessary to determine the optimum dosage in batch experiments for any wastewater. Low PAC dosages (in the neighbourhood of 0.5 g/L) coupled with short SRTs have been recommended for membrane fouling mitigation in MBRs [144]. The optimum dosage of the adsorbents will also allow the striking of a balance between the cost savings arising from membrane fouling abatement and the cost of the additives and handling of the resulting sludge.

### 4.3. Use of Granular Biomass (Aerobic Granulation)

As indicated in Section 3.3.3, EPS are the construction materials for microbial aggregates. As such, recent research has focused on incorporating biotechnological processes that can use up these foulants. A key innovation in this regard is the integration of aerobic granulation biotechnology with MBR to develop the aerobic granulation membrane bioreactor (AGMBR) as a novel approach to control membrane fouling. Aerobic granulation refers to the process of microbe-to-microbe self-immobilisation without any biocarriers [145,146,147,148]. The resulting granular biomass are dense microbial consortia packed with different microbial species that can collectively degrade wastewater pollutants [77,149]. Compared to the ASP, aerobic granulation technology offers the following advantages: excellent settling properties, smaller footprints, strong microbial structure, higher biomass retention, less sludge production, high resilience to toxic chemicals, and good ability to handle high organic and shock loading rates [77,111,149,150,151,152]. AGMBR offers a distinctive advantage of utilising EPS for granule formation and offering the large size and rigid structure of the granules for bacteria to attach to rather than the membrane surface. The large size and rigid structure of the granules is expected to reduce cake-layer formation, pore blocking and surface deposition on the membrane surface.

The integration of aerobic granulation and MBR was first reported by Li *et al.* [112]. Findings from their study showed that membrane permeability in the AGMBR system was over 50% higher when compared to conventional MBR. Following this was a four-months bench-scale study by Tay *et al.* [11] who compared the processes and performances of AGMBR and submerged MBR. They found that while AGMBR and submerged MBR showed similar treatment efficiencies, the AGMBR showed much better filtration characteristics with the membrane permeability loss (34.5%) in AGMBR being twice as low as that of submerged MBR at constant pressure testing. Constant flux testing also showed that, increasing the flux threefold resulted in membrane permeability loss of 2.4% with the AGMBR mixed liquor (*i.e.*, 21 times lower than that of the MBR). In continuous operational mode, the TMP increment in the AGMBR was negligible (3–6 kPa) and the membrane required no physical cleaning; whereas, the TMP increment in the submerged MBR was significant (50–60 kPa) and regular physical cleaning of the membrane module was required. Similarly, in a long-term study (10 months), Tu *et al.* [18] reported a higher removal efficiency of pollutants, as well as improved membrane performance (fouling rate was maintained below 0.1 kPa/day at MLSS > 18,000 mg/L) when aerobic granules were formed in MBR. They indicated that the granule size change and improved settling ability were responsible for maintaining membrane permeability.

Another study by Juang *et al.* [105] investigated membrane fouling in AGMBR and reported that most bacteria cells were retained by the granules thus preventing their penetration through the membrane pores and the chance to cause internal fouling layer. The combination of aerobic granulation and MBR has also been reported to enhance good filtration performance and lower propensity for membrane fouling [115]. Other reported findings on AGMBR include: stable operation at 20 L/m^2^ h for 61 days with significant filtration improvement [153]; extension of filtration period by 78 days without physical cleaning [111]; and excellent fouling control [154].

Regarding treatment performance, aerobic granulation offers diverse microbial aggregates that exhibit superior treatment efficiency when compared to floccular sludge. This is attributable to the strong microbial structure of aerobic granule as well as their high biomass retention and high resilience to toxic chemicals. Tu *et al.* [18] reported a higher removal efficiency of pollutants when aerobic granules were formed in MBR. Aerobic granules exhibit a layered structure with an oxic zone near the granule surface, an anoxic zone in the middle layer, and an anaerobic core at the granule centre [155,156]. This layered structure is suitable for simultaneous organics, nitrogen, and phosphorus removal. A study conducted to determine the removal of nitrogen in AGMBR reported about 60% total nitrogen removal in the AGMBR [111]. Another study to determine the performance of AGMBR reported the removal of COD, total phosphorus, nitrate-nitrogen and total nitrogen as 93.17%, 90.42%, 95% and 95%, respectively [157].

However, the major technical problem of AGMBR is the long-term system operation instability of aerobic granulation and granule disintegration problems [151,158]. Aerobic granules have been observed to disintegrate after prolonged operation [149,159,160,161]. The deterioration in granule stability over time impacts the efficiency of wastewater treatment and is a major issue affecting the effectiveness of aerobic granulation in full-scale operations. As applied to AGMBR, the granule disintegration increases the concentration of soluble EPS, consequently increasing the membrane fouling propensity [154]. Hence, the production of granules with sustainable long-term structural integrity is a key area requiring further research.

### 4.4. Use of Granular Materials with Aeration

To enhance the detachment of foulants from the membrane in MBR, research has focused on using granular materials with air scouring to provide continuous mechanical cleaning. In this regard, Siembida *et al.* [162] reported that the abrasion produced by granular materials introduced into the MBR tank significantly reduced cake layer formation on the membranes. The study further found that this method resulted in a successful long-term operation (more than 600 days) at permeate flux of 40 L/m^2^ h without membrane chemical cleaning. The introduction of the granular materials also allowed MBR operation at a higher permeate flux (more than 20% higher) compared to the conventional MBR. Similarly, Kurita *et al.* [163] found that the introduction of granular materials (made of polyethylene glycol) into a submerged MBR increased the critical flux by more than 40%; allowed for the stable operation of MBR despite the reduction of aeration by 50%. The reduction of aeration would remarkably reduce MBR operational and maintenance cost. Johir *et al.* [164] studied the effect of different particle sizes of granular activated carbon (GAC) on submerged MBR operated at a filtration flux of 20 L/m^2^ h. Three size ranges of GAC were used in the study: 150–300, 300–600 and 600–1200 μm. The authors reported that GAC size played a significant role in membrane fouling abatement as the total membrane resistance reduced by 60% with GAC particle sizes of 300–600 μm. In addition, organics removal was up to 95% with the addition GAC.

Furthermore, Pradhan *et al.* [165] found that the addition of granular media in the MBR reactor resulted in the same TMP reduction as doubling the aeration intensity (from 600 to 1200 L/h/m^2^). A recent study also found that the rate of aeration in MBR can be reduced by over 50% with the introduction of granular materials [166]. In the same regard, Krause *et al.* [167] investigated the removal of the membrane fouling layer by continuous physical abrasion by adding granular materials to the activated sludge. They reported no membrane permeability decline throughout the over eight months of operation at fluxes of up to 40 L/m^2^ h.

The effectiveness of using granular material as a fouling control mechanism in anaerobic fluidised membrane bioreactor (AFMBR) has also been reported in the literature. Kim *at al.* [168] examined the effect of placing GAC directly in contact with membranes in an AFMBR in a 120-day continuous-feed experiment using two-stage anaerobic treatment system: first stage consisting of a fluidised-bed bioreactor without membranes followed by an AFMBR. The fluidised GAC produced scouring action on the membrane surface which reduced membrane fouling evidenced by the cleaning of the membrane only twice during the 120 days of operation. The authors reported an energy requirement of 0.028 kWh/m^3^, which is remarkably low compared to the values reported for anaerobic membrane bioreactors using gas sparging for membrane fouling control. Similarly, Aslam *et al.* [169] studied the effectiveness of using GAC and non-adsorbing silica and polyethylene terephthalate (PET) beads as fluidised media in an AFMBR in reducing membrane fouling and for energy requirement for fluidisation. Findings of their study indicated that GAC can reduce membrane fouling both by adsorption of foulants and scouring action along membrane surfaces. Smaller particles demonstrated higher sorption capacity and less energy requirement for fluidisation until sorption capacity was exhausted. Afterwards, membrane scouring became the dominant mechanism; and, fouling reduction was a function of energy expenditure, with larger GAC particles that required more energy for fluidisation providing the best fouling reduction. In addition, increasing the packing ratio of GAC particles from 10% to 50% increased the energy required for fluidisation and also lowered the membrane fouling rate. Non-adsorbing silica particles and PET beads demonstrated similar results to pre-adsorbed GAC, lower fouling was accomplished by the larger media that had a higher energy requirement for fluidisation. Fouling reduction was also somewhat better at a given energy expenditure with lower specific gravity PET beads than with denser and smaller pre-adsorbed GAC particles. Additionally, recently, Kim *et al.* [170] used GAC as the fluidised particles for scouring the membrane and as a support for bacterial growth. They found that membrane fouling was successfully controlled by GAC fluidisation as the TMP was low, and could be maintained below 0.12 bar by a daily removal of excess solids. With the scouring action of the GAC and daily withdrawal of solids, membrane cleaning was not required. These results open up a new possibility of using this method to make chemical cleaning of the membrane a seldom activity in MBR operation. However, granular materials have a tendency to damage the membrane material. Further research is needed to find the optimum aeration intensity and the right granular material that would not cause damage to the membranes.

### 4.5. Quorum Quenching

Another innovative approach to membrane fouling control in MBRs is the use of quorum quenching. The regulation of microbial group behaviours by cell-to-cell communication (quorum sensing) is involved in biofilm formation [171]. Bacteria produce autoinducers, which are used in intercellular communication [172]. When the concentration of autoinducer attains a threshold level, it combines with the receptor protein and activates the transcription of specific genes to induce group behaviours such as biofilm formation and EPS production [171,173]. On this basis, the concept of quorum quenching was introduced in MBR for biofouling control through quorum sensing (QS) control by the blocking intercellular communications.

Yeon *et al.* [174] utilised acylase attached to magnetic carrier to inhibit QS in MBR; and, they reported reduced biofouling and enhanced the membrane permeability. Another study found that disrupting the energy metabolism and subsequently producing QS signaling molecules effectively controlled biofouling [175]. Similarly, Jahangir *et al.* [176] and Oh *et al.* [177] investigated membrane biofouling control by encapsulating quorum quenching bacteria into a hollow fiber membrane. Findings from these two separate studies showed that membrane biofouling was successfully inhibited. The study of Jahangir *et al.* [176] further found that as the recirculation rate (of the mixed liquor between the bioreactor and the membrane tank) increased, the biofouling inhibition (quorum quenching effect) increased. This was attributed to the facilitated transport of signal molecules from the biofilm into the bulk mixed liquor, and then to the microbial-vessel.

Kim *et al.* [178] immobilised acylase onto the membrane surface and reported that the MBR system showed mitigation of membrane biofouling. Similarly, Jiang *et al.* [173] immobilised acylase into sodium alginate capsules for enzymatic quorum quenching in MBRs. They reported that quorum quenching influenced sludge characteristics and membrane biofouling through better sludge settleability, smaller sludge particle size, less production of EPS and SMPs, lower apparent viscosity and higher zeta potential of mixed liquor. Quorum quenching further altered the characteristics, behaviour and function of EPS and SMPs, ultimately weakened biofilm formation ability but enhanced membrane filterability.

Another quorum quenching approach reported in the literature is the use of free-moving beads entrapped with quorum quenching bacteria [179]. The cell entrapping beads (CEBs) were prepared by entrapping quorum quenching bacteria (*Rhodococcus* sp. BH4) into alginate beads. The introduction of CEBs in the MBR significantly delayed the TMP rise (the time to reach a TMP of 70 kPa was 10 times longer than the control MBR). This membrane biofouling mitigation was attributed to both physical (abrasion) and biological (quorum quenching) effects of CEBs. The explanation is that microbial cells in the biofilm produced fewer EPS and thus formed a loosely bound biofilm due to the quorum quenching effect of CEBs. This enabled the biofilm to slough off easily from the membrane surface from the abrasion of the CEBs.

Additionally, recently, Wood *et al.* [180] engineered a beneficial biofilm that mitigates biofouling (through limiting its own thickness) by sensing the number of its cells that are present via a QS circuit. This was based on the secretion and uptake of a communication signal; hence, biofilm formation was limited by self-monitoring and selective dispersal. The beneficial biofilm prevented the formation of biofilm by deleterious bacteria through the secretion of nitric oxide, a general biofilm dispersal agent. The engineered beneficial biofilm further removed the environmental pollutant, epichlorohydrin, through the production of epoxide hydrolase. Thus, the engineered beneficial biofilm plays two significant roles: mitigation of membrane biofouling and provision of a platform for biodegradation of recalcitrant organic pollutants.

However, practical issues on the cost and stability of enzymes that are used in QS are yet to be determined. The use of free-moving beads entrapped with quorum quenching bacteria can open the door to practical application but this quorum quenching method needs pilot-scale testing. Engineered living biofouling-resistant membrane system needs further testing at pilot-scale.

## 5. Conclusions

In this paper, fundamentals of membrane fouling and advances in fouling mitigation strategies in MBRs are reviewed. Membrane fouling in MBRs can be classified into biofoulants, organic foulants and inorganic foulants based on their biological and chemical characteristics. Of these, biofoulants and organic foulants are the major contributors to membrane fouling in MBRs. Most research on membrane fouling targets these foulants. There are different factors that influence membrane fouling in MBRs. These factors include: membrane characteristics (material type, water affinity, surface roughness, surface charge, and pore size), operating conditions (operating mode, rate of aeration, SRT, HRT, F/M ratio, OLR, COD/N ratio, and temperature), and feed and biomass characteristics (MLSS, sludge apparent viscosity, EPS, floc size, alkalinity, pH and salinity). EPS, in particular, are major contributors to membrane fouling.

Current research trends for membrane fouling mitigation in MBR have been presented; namely, the addition of coagulants and adsorbents, use of granular biomass, use of granular materials with air scouring, and quorum quenching. The addition of coagulants and adsorbents shows significant membrane fouling reduction but further research is needed to establish optimum dosages of the various coagulants and adsorbents in order to strike a balance between cost savings arising from fouling abatement and the cost of the additives and handling of the resulting sludge. Aerobic granulation is a promising biotechnology that targets biofouling and organic fouling. Early results of integrating aerobic granulation with MBRs show significant reduction in membrane fouling as well as enhanced organics and nutrients removal. However, AGMBR is still in the development phase. Further research is needed to establish the EPS-membrane fouling relationship in AGMBR and optimum operating conditions on real wastewater applications. Granule stability and disintegration in long-term operation is a major shortcoming of the granulation technology, which brings forth an area for further research. The introduction of granular materials with aeration in the MBR tank to provide continuous mechanical cleaning significantly reduces the cake layer formation, resulting in successful long-term operation. Quorum quenching also offers a strong potential for fouling control but pilot-scale testing is required to explore the feasibility of full-scale application.

## Figures and Tables

**Table 1:** Factors affecting membrane fouling in membrane bioreactors (MBR).

Factor	Effect on Membrane Fouling	Reference
**Membrane Characteristics**		
Membrane Material	Ceramic membranes are hydrophilic, hence they foul less. Polymeric membranes are mostly hydrophobic and exhibit more fouling	[5,13,41,42]
Water affinity	Increasing hydrophilicity indicates less membrane fouling propensity while hydrophobicity correlates well with increase propensity for membrane fouling	[47]
Membrane surface roughness	Membrane fouling tends to increase with increasing surface roughness as the rough surface provides a valley for the colloidal particles in the wastewater to accumulate on. However, higher projections on the membrane surface exhibit higher antifouling property and better permeability recovery after backflushing than gentle roughness.	[48,49,50,51]
Membrane surface charge	The colloidal particles depositing on the membrane makes them negatively charged, hence they can attract cations in the MLSS, such as Ca^2+^ and Al^3+^ leading to inorganic fouling	[49]
Membrane pore size	Increasing membrane pore size increases the tendency for pore blocking mechanism	[47,53]
**Operating Conditions**		
Operating mode	Operating in cross-flow filtration mode reduces cake layer formation on the membrane surface	[69]
Aeration	Increasing aeration rates results in a reduction in membrane fouling	[59,60,61]
Solids retention time (SRT)	Operating at high SRTs reduces the production of EPS, hence reduced fouling. However, extremely high SRTs rather increase membrane fouling due to the accumulation of MLSS and increased sludge viscosity	[65,66,67,68]
Hydraulic retention time (HRT)	Decreasing HRTs results in increasing rate of membrane fouling. However, extremely high HRTs leads to an accumulation of foulants	[30,71,72,73,74]
Food-microorganisms (F/M) ratio	The rate of membrane fouling in MBRs increases with increasing F/M ratio due high food utilisation by biomass resulting in increased EPS production	[70,75,76]
Organic loading rate (OLR)	Membranes foul more as OLR increases	[79]
COD/N ratio	Operating at higher COD/N ratio reduces rate of membrane fouling, improved membrane performance and a longer operation period before membrane cleaning	[81,82]
	On the contrary, other studies found that low COD/N ratio results in lower MLSS concentration, lower SMPs production, lower carbohydrates, proteins, and humic acids in LB-EPS; hence, low membrane fouling	[83,84,85]
Temperature	Low temperatures increase the propensity for membrane fouling as more EPS are released by bacteria and the number of filamentous bacteria increases. Sudden temperature changes also increase fouling rate due to spontaneous release of SMPs	[30,86,88,89]
**Feed/biomass characteristics**		
Mixed liquor suspended solids (MLSS)	Increasing MLSS correlate with increased rate of membrane fouling	[60,61,90,91]
	Other studies report no (or little) effect of MLSS on membrane fouling	[93,94,95]
Sludge apparent viscosity	Increasing the viscosity results in increased membrane fouling	[60,97]
Extracellular polymeric substances (EPS)	Increase in the concentration of EPS (bound EPS and SMPs) result in membrane fouling	[2,36,103,104]
Floc size	Decrease in floc size increases membrane fouling	[109]
pH	Decrease in pH results in increased rate of membrane fouling	[116,118,119]
Salinity	Increasing salinity increases membrane fouling by altering biomass characteristic like more release of bound EPS and SMPs, floc size and zeta potential	[121,122,123]

## Abbreviations

The following abbreviations are used in this manuscript:

AFMBR	anaerobic fluidised membrane bioreactor
AGMBR	aerobic granulation membrane bioreactor
ASP	activated sludge process
BAP	biomass-associated products
BPC	biopolymer clusters
CA	cellulose acetate
CAB	cellulose acetate butyrate
CEBs	cell entrapping beads
COD	chemical oxygen demand
DO	dissolved oxygen
EPS	extracellular polymeric substances
FeCl_3_	ferric chloride
F/M	food-microorganisms
GAC	granular activated carbon
HRT	hydraulic retention time
IUPAC	International Union of Pure and Applied Chemistry
LB-EPS	loosely bound extracellular polymeric substances
MLSS	mixed liquor suspended solids
MBR	membrane bioreactor
MF	microfiltration
NF	nanofiltration
OLR	organic loading rate
PAC	powdered activated carbon
PACl	polymeric aluminium chloride
PAFC	polymeric aluminium ferric chloride
PAN	polyacrylonitrile
PE	polyethylene
PES	polyethersulfone
PET	polyethylene terephthalate
PFS	polymeric ferric sulphate
PP	polypropylene
PS	polysulfone
PTFE	polytetrafluoroethylene
PVB	polyvinyl butyral
PVDF	polyvinylidene fluoride
QS	quorum sensing
RO	reverse osmosis
SMPs	soluble microbial products
SRT	solids retention time
SVI	sludge volume index
TB-EPS	tightly bound extracellular polymeric substances
TMP	transmembrane pressure
TOC	total organic carbon
UAP	utilisation-associated products
UF	ultrafiltration
